# Physical activity levels and psychological parameters among university students following the COVID-19 pandemic

**DOI:** 10.1186/s13102-025-01166-7

**Published:** 2025-05-08

**Authors:** Ahmet Yapar, Can Özgider, İlhan Adiloğulları, Özhan Bavlı, Gamze Elif Adiloğulları

**Affiliations:** Faculty of Sports Sciences, Canakkale Onsekiz Mart University, Canakkale, Turkey

**Keywords:** Physical activity, Psychological resilience, Self-confidence, Self-efficacy, College students

## Abstract

**Background:**

The worldwide pandemic caused by the novel coronavirus has profoundly impacted virtually every aspect of life. The education sector was also significantly impacted, with numerous educational institutions adopting online learning due to the pandemic. The university period is one of substantial transformation and transition for young individuals. During this educational stage, the advent of emerging social networks, coupled with the necessity for effective network management, can precipitate stress in university students, potentially leading to alterations in their psychological well-being. The objective of this study was to compare the self-efficacy, psychological resilience and self-confidence of university students with different levels of physical activity (PA) according to gender and school year variables.

**Methods:**

The study was a cross-sectional design. The Sample comprised 2,868 undergraduate students, 1,405 female and 1,463 male, enrolled in 10 different faculties at Çanakkale Onsekiz Mart University. The participants were administered the International Physical Activity Questionnaire, the General Self-Confidence Scale, the Psychological Resilience Scale and the Self-Efficacy Scale.

**Results:**

Significant difference was observed between the general self-efficacy score of participants with low and moderate PA levels and those with high levels of PA. The psychological resilience variable was affected by the level of PA. Individuals with a low level of PA exhibited a lower level of psychological resilience than those with a moderate or high level of PA. Upon analysis of self-confidence, both internal and external self-confidence scores demonstrated an upward trajectory for the low, moderate, and high PA groups. Furthermore, the results of the study indicated that as the level of PA increased, there was a corresponding increase in self-efficacy, psychological resilience and self-confidence among university students.

**Conclusions:**

Results indicated that higher levels of PA among university students were positively associated with increased self-confidence, self-efficacy, and psychological resilience. Consequently, by facilitating the organization of diverse physical activities and providing opportunities, university administrations can foster enhanced self-confidence, self-efficacy and psychological resilience among their students.

**Supplementary Information:**

The online version contains supplementary material available at 10.1186/s13102-025-01166-7.

## Introduction

The global pandemic of the novel coronavirus, which has lasted for more than three years, has had a profound effect on all aspects of life. The education sector was also significantly affected, with many educational institutions adopting online learning because of the pandemic. It was posited that online education, while offering numerous benefits, has simultaneously imposed numerous restrictions on students, resulting in a range of adverse social, physical, and mental effects, as well as simultaneously increasing stress levels and negatively affecting their general well-being [1]. A substantial body of scientific literature exists on this topic, with numerous studies conducted to investigate these effects, including one that has investigated the psychological resilience, stress, and physical activity (PA) of university students during the pandemic period [2]. Similarly, relationships among anxiety, depression, and PA were examined [3]. Another study examined the relationships among PA, psychological resilience, mood, emotions, and weight control [4]. Furthermore, the psychological effect of the COVID-19 pandemic was investigated [5], and the psychological resilience of individuals during a pandemic was explored [6].

Following the pandemic, numerous countries relaxed long-term restrictions and initiated a period of normalization [7]. During this process, university administrations were required to implement an effective management approach to address the challenges and changes associated with the post-pandemic period [8]. University students were subjected to numerous restrictions and online learning for an extended period because of the pandemic. Consequently, their needs, including the desire to engage in PA, were not fully addressed in the post-pandemic period [9]. This resulted in a decline in their well-being and increased levels of stress and depression [10, 11]. Because of this state of mind, individuals may become susceptible to a negative emotional disposition, which may in turn impinge upon their capacity to succeed [12]. As evidenced by research in exercise and sport psychology, participation in PA represents a crucial aspect of leading a healthy lifestyle [13, 14]. This participation has been shown to elicit positive emotional experiences among university students and mitigate the negative emotions of anxiety and depression to some extent [15]. Additionally, studies have indicated that participation in physical activities such as running and cycling can be an effective means of alleviating anxiety, depression, and other stressful situations [16]. In other words, the necessity for PA in the post-pandemic context is critical for university students to lead healthy lives and actively address challenges to sustain their well-being. So, participation in PA not only fulfills the PA needs of university students but also helps them recover from the psychological tension caused by the pandemic. This, in turn, helps them remain satisfied and full of expectations for their future education [17].

In addition to preventing a range of noncommunicable diseases (NCDs), such as cardiovascular disease and type 2 diabetes [18], numerous studies have demonstrated the positive effects of PA on mental health and cognitive function, particularly depression [19, 20]. The participation of students in PA, their status regarding PA, and feelings about their psychological and emotional state are important issues. However, the issue of healthy behaviour and healthy living remains on the list of things to do among emerging adults [21, 22]. This lack of attention can result in a reduction in physical exercise, an increased tendency to skip a healthy breakfast at home, and an increase in smoking, excessive alcohol consumption, and the use of other banned substances [23]. Conversely, initiatives such as healthy campuses are emphasized by public and university administrations and are important during the transition from high school to the first year of university. Indeed, numerous studies have demonstrated that participation in PA decreases during this period [24–26].

Psychological resilience is a positive resource, defined as an enhanced and positive capacity to recover from challenges, conflicts, and failures [27]. Three main groups of protective factors related to resilience have been identified: family connectedness (e.g., parental support, communication skills), social resources (e.g., community support, environment, social activity), and personal dispositions (e.g., individual characteristics, self-regulation, self-esteem) [28, 29]. PA has gained prominence as a protective factor in facilitating the recovery of adolescents from the mental health challenges they encountered during the global health crisis of the COVID-19 pandemic [30]. There is a growing body of evidence suggesting that PA can lead to positive psychological outcomes, with adolescents who engage in PA displaying higher levels of psychological resilience [31]. Similarly, in both Norwegian and Chinese samples, adolescents who engaged in frequent PA presented higher scores on protective factors related to psychological resilience than did their less physically active counterparts [32, 33]. A recent randomized controlled trial demonstrated that PA-based psychological resilience notably increased during the period of prevention and control of the COVID-19 pandemic [34]. Despite the likelihood of negative emotional responses following stressful or traumatic events (e.g., the COVID-19 pandemic), individuals with high psychological resilience may demonstrate more retained empathy and problem-solving abilities. This implies that those with higher levels of resilience are capable of self-reflection, possess proficient social skills, and exhibit compassion toward others [35]. These attributes are of paramount importance for effective interpersonal adjustment. With the advent of activity psychology, the positive mental health benefits of PA have been widely acknowledged [36, 37]. Among these beneficial effects, PA plays an essential role in enhancing an individual’s sense of efficacy [38]. Individuals demonstrate enhanced self-efficacy following vigorous jogging or cycling [39]. The study revealed that the quantity of PA has a considerable influence on the academic mood and self-efficacy of high school students. Moreover, the effects of different intensities of PA on self-efficacy are significantly distinct [40]. A further significant relationship was identified between PA and self-efficacy in university students, whereby the higher the degree of PA is, the greater the self-efficacy [41]. Furthermore, numerous studies have identified a strong correlation between PA and self-confidence. For example, it was demonstrated that self-efficacy plays a pivotal role in influencing PA through the implementation of self-management strategies [42]. This finding indicates that the thoughts, goals, plans, and actions that facilitate PA are contingent upon self-efficacy. Similarly, in another study it was reported that self-efficacy, which was defined by Bandura [43] as an individual’s belief in their ability to perform behaviours necessary to achieve specific outcomes, is a dominant correlate of active engagement in PA [44]. Moreover, the existing literature contains studies that indicate a correlation between the levels of PA and self-esteem in samples comprising adolescents, young people and adults. Amimita-Badestamon et al., [45] implied that the level of PA increases the level of self-esteem significantly, which means that encouraging regular PA could be a valuable strategy for promoting positive self-esteem among junior high school students. Besides, in a study it was demonstrated that self-efficacy mediates the relationship between motivation and PA in patients with heart failure, emphasizing the significance of self-efficacy in PA [46]. Furthermore, detailed studies have showed a relationship between self-efficacy and PA [47, 48]. Studies have demonstrated that self-efficacy has a direct and indirect influence on PA levels through the factors of goals, social support, fatigue, and outcome expectations. This emphasizes the multifaceted role of self-efficacy in PA participation. Moreover, it was reported that a moderate amount of PA was positively associated with higher self-esteem levels [49]. This further corroborates the correlation between PA and self-esteem. Collectively, the literature substantiates the association between PA level and self-confidence, with self-efficacy playing a pivotal intermediary role. These outcomes underscore the significance of self-efficacy and confidence in promoting and maintaining PA involvement. The university years are seen as a transformative period marked by significant academic, social, and personal changes [50]. Students face heightened academic expectations, requiring greater responsibility and self-directed learning, which can create stress. At the same time, adapting to new social environments and forming relationships can impact psychological well-being. These challenges were further intensified by the COVID-19 pandemic, which disrupted traditional learning, increased isolation and anxiety. While this period offers opportunities for growth, the combined impact of academic pressure, social adjustment, and pandemic-related uncertainties has significantly affected students’ overall well-being and success [51].

Considering these findings, there is a need to examine the university students’ PA levels and psychological parameters during the post-COVID-19 pandemic. This research aims to address the existing gaps in the understanding of the effects of the pandemic on the psychological well-being of university students and the role of PA in alleviating psychological distress. The purpose of this study was to compare the self-efficacy, psychological resilience and self-confidence of university students with different levels of PA according to gender and school year variables.

## Method

A cross-sectional design was employed in this study, which is one of the survey research methods [52, 53]. The survey research method is used to ascertain the characteristics of a group. The objective of cross-sectional studies is to identify the status of the investigated characteristics of the group observed via the survey method over a specified time interval [54].

### Participants

The study population comprises undergraduate students enrolled at Çanakkale Onsekiz Mart University during the 2021–2022 academic year. The inclusion criteria for the study were as follows: Participants had to be active students enrolled in one of the departments at Çanakkale Onsekiz Mart University, being between the ages of 17 and 30, have no physical health issues, and voluntarily agree to participate in the study. The sample size of the study was 2.868 undergraduate students, of which 1.463 were males and 1.405 were females, all between the ages of 17 and 30 (mean = 20.07). They were selected from 10 out of 21 faculties of Çanakkale Onsekiz Mart University using the stratified sampling method. The stratified sampling method is a technique employed when examining variables that vary according to a particular characteristic of the study participants (e.g., age, gender, socioeconomic status, and cultural background) [55]. This method allows for the estimation of separate parameters for each of the identified stratifications [56]. Table 1 illustrates the demographic distribution of the gender and age of the participants according to their academic faculties.

**Table 1:** Descriptive information of mean age and gender of the students by their faculties

Faculty	Female			Male		
	*n*	%	Average Age	*n*	%	Average Age
Medicine	25	1.8	22.52	25	1.7	22.08
Business Administration	73	5.2	19.81	126	8.6	19.69
Engineering	163	11.6	20.56	481	32.9	19.97
Science and Literature	160	11.4	19.45	125	8.5	20.31
Agriculture	82	5.8	19.21	194	13.3	19.45
Sport Sciences	121	8.6	20.60	129	8.8	20.76
Health Sciences	435	31.0	19.48	176	12.0	19.98
Political Sciences	106	7.5	20.37	93	6.4	20.71
Fine Arts	89	6.3	20.97	24	1.6	20.46
Architecture	151	10.7	20.03	90	6.2	20.34
Total	1405	100.0		1463	100.0	

### Data collection procedures

The objective of this project was to be initiated during the 2019–2020 academic year. However, due to the onset of the COVID-19 pandemic in Turkey in March 2020, educational institutions were compelled to transition from face-to-face to online (distance) learning modalities. Initially, the data collection was planned to be conducted in person with potential participants (i.e., university students) from our institution. However, the transition to online education necessitated by the pandemic rendered this approach unfeasible. Consequently, the data collection was postponed until the resumption of in-person education.

In this context, the data for this study were collected by the researchers during the spring semester of the 2021–2022 academic year. This was accomplished via several tools, which were applied in a face-to-face manner. Once the participants had been informed about the research and the data collection tools, they were distributed to the volunteer participants. The questionnaires were completed in around 10 min.

### International physical activity questionnaire (Short Form)

The questionnaire was employed to ascertain the levels of physical activity (PA) engaged in by the participants. The cross-national validation and reliability studies of the questionnaire were conducted by Craig et al. [57] and subsequently adapted into Turkish by Öztürk [58] before being designated the International Physical Activity Questionnaire (IPAQ) short form. The questionnaire is recommended for use with adults aged between 18 and 69 years. The reliability was tested Savcı et al., [59] by using an accelerometer. Test-retest of the Turkish version of the IPAQ short form were reported as *r* = 0.30 and 0.69. The questionnaire includes a series of inquiries about the frequency and duration of PA undertaken for a minimum of 10 min over the previous seven days [60, 61].

IPAQ–SF is composed of 7 open-ended questions that measure the types (Vigorous, Moderate, Walking and Sitting) and times (Days per week, hours and minutes per day) of PA of participants. The types and time of physical activities were converted to metabolic equivalent task minutes per week (MET-min/week) following the IPAQ-SF scoring protocol. In this protocol, computation of MET-minutes for the Leisure domain is calculated by the formula as listed below:

- Walking MET-minutes/week leisure = 3.3 * walking minutes * walking days in leisure.
- Moderate MET-minutes/week leisure = 4.0 * moderate-intensity activity minutes * moderate-intensity days in leisure.
- Vigorous MET-minutes/week leisure = 8.0 * vigorous-intensity activity minutes * vigorous-intensity days in leisure.
- Total Leisure-Time MET-minutes/week = Sum of Walking + Moderate + Vigorous MET-minutes/week scores in leisure.

According to the IPAQ-SF guideline, PA scores are categorized as Low, Moderate and High. These categories are determined by the calculation of PA time, days of PA and MET scores. The categorization of MET scores is listed below:

- Low PA Level < 600 MET-minutes/week.
- Moderate PA Level 601–2999 MET-minutes/week.
- Vigorous PA Level > 3000 MET-minutes/week.

### Self-confidence scale

The 33-item self-confidence scale [62] was employed to evaluate the participants’ self-confidence levels. The scale is a five-point Likert-type scale comprising two subdimensions: intrinsic self-confidence and extrinsic self-confidence. The internal consistency coefficients of the self-confidence scale were found to be 0.83 for the entire scale and 0.83 and 0.85 for the internal self-confidence and external self-confidence subdimensions, respectively. The items comprising the internal self-confidence subdimension are as follows: items 4, 25, 32, 17, 10, 30, 12, 3, 19, 5, 21, 27, 9, 23, 1, 7, 15. The items forming the external self-confidence subdimension are items 6, 31, 20, 29, 16, 14, 22, 11, 18, 33, 2, 28, 26, 13, 8 and 24, which collectively account for 43.6% of the total variance.

### Self-efficacy scale

The General Self-Efficacy Scale [63] was adapted into the Turkish language [64] as the Generalized Self-Efficacy Expectancy Scale. The scale, which was developed to assess general self-efficacy, aims to predict an individual’s capacity to cope with daily challenges and adapt following exposure to a range of stressful experiences. This scale is suitable for people who is over 12 years old. The scale employs a four-point Likert-type format, comprising a total of 10 items. The responses to each item are summed to obtain a single score, with a range of 10–40. A high score indicates a positive perception of self-efficacy, whereas a low score indicates a negative perception. In the original study, the Cronbach’s alpha internal consistency coefficient of the scale was 0.82. The Cronbach’s alpha internal consistency coefficient of the scale was 0.83. In this study, the Cronbach’s α internal consistency coefficient of the scale was 0.90.

### The brief resilience scale (BRS)

To assess the psychological resilience of the participants, the brief resilience scale (BRS) [65] and subsequently translated into the Turkish language [66], was employed. The BRS is a one-dimensional scale comprising six items presented in a 5-point Likert format. Following a confirmatory factor analysis, the scale exhibited a single-factor structure. The internal consistency coefficient of the BRS was found to be 0.83.

### Data analysis

The data gathered during the research were initially entered into the data analysis software (IBM SPSS 21.0). The data about the demographic characteristics of the participants were subsequently subjected to descriptive statistical analysis. To perform the inferential statistical analyses, normality tests of the data were conducted via the Kolmogorov‒Smirnov test, whereas the skewness and kurtosis test was employed to determine the skewness and kurtosis values. One-way ANOVA was utilized for data sets comprising more than two groups with a normal distribution, whereas the Kruskal‒Wallis and Mann‒Whitney U tests were employed to analyze data sets that did not demonstrate a normal distribution.

### Data cleaning and missing data treatment

The missing cases were utilizing listwise deletion. In this method, cases with missing scores were excluded from the study to ensure internal consistency of the dataset for the analysis.

### Ethics

Prior to data collection, ethical approval for this study was granted by Canakkale Onsekiz Mart University Clinical Research Ethics Committee on 31 October 2018, with decision number 19 − 2.

### Findings

#### Demographic characteristics of the participants

**Table 2:** Physical activity levels of students by gender and school year variable

		Female			Male		
School Year	Physical Activity Level*	*n*	%	MET_median_	*n*	%	MET_median_
Preparatory	Low	75	21.0	139	74	24.3	128
	Moderate	182	51.0	1413	134	44.1	1473
	High	100	28.0	4269	96	31.6	4578
1^st^ Year	Low	77	13.2	181	97	13.4	191
	Moderate	313	53.5	1626	276	38.0	1759
	High	195	33.3	4479	353	48.6	4770
2^nd^ Year	Low	34	12.7	243	20	10.4	120
	Moderate	118	44.0	1386	81	42.0	1140
	High	116	43.3	4237	92	47.7	5304
3^rd^ Year	Low	25	14.1	160	45	20.6	132
	Moderate	74	41.8	1645	77	35.3	1485
	High	78	44.1	4693	96	44.0	5118
4^th^ Year	Low	3	16.7	480	2	10.0	74
	Moderate	5	27.8	2376	7	35.0	1786
	High	10	55.6	5688	11	55.0	4746

*Low: MET minutes range ≤ 600); Moderate: MET minutes range: ≥601 and ≤ 2999; High MET minutes range ≤ 3000

Table 2 presents the physical activity (PA) levels of students based on their gender (female and male) and academic year (Preparatory to 4th Year). PA was categorized into three levels: low, moderate, and high, and the data includes the number of students (n), the percentage within each group, and the median MET (Metabolic Equivalent of Task) values for each level.

In the Preparatory year, most of both female (51.0%) and male (44.1%) students fell under the moderate activity level. However, a significant portion of students remained in the low level activity category (21.0% of females and 24.3% of males). Interestingly, male students showed slightly higher MET medians in both the moderate and high levels of PA when compared to females.

Among 1st-year students, there was a noticeable shift toward higher activity levels, particularly among males, where 48.6% showed a high PA level compared to 33.3% of females. Both genders exhibited increased MET medians at higher activity levels.

2nd-year students continued this trend, with high activity levels becoming more dominant; 43.3% of females and 47.7% of males indicated high-level activity. MET medians were higher in males than in females across all levels, especially in the high-level activity group.

In the 3rd year, the distribution became more balanced. Approximately 44% of both male and female students were in the high-level activity group, though the percentage of males in the low activity level increased slightly to 20.6%. MET medians remained relatively high in both genders at the high activity level.

For 4th-year students, the proportion of those engaging in high PA reached its peak, with 55.6% of females and 55.0% of males categorized as highly active. MET values also peaked at this level, indicating the highest energy expenditure in both genders.

Overall, the data showed a clear progression in PA levels as students advance through their academic years, with a general increase in both the percentage of students in the high-level activity category and their corresponding MET medians. This trend was evident in both male and female groups, although males tended to consistently report slightly higher MET values.

### Comparison of general self-efficacy according to physical activity level, gender and school year variables

Based on the results of the international physical activity-short form, the participants’ PA status was classified as low (MET minutes ≤ 600), moderate (601 ≤ MET minutes ≤ 2999), or high (MET minutes ≥ 3000), according to the corresponding MET values.

An examination of the scores assigned by the participants to the general self-efficacy scale revealed that the scale did not meet the assumption of a normal distribution (skewness = 0.581, standard error = 0.046, kurtosis = 4.209, standard error = 0.092; *p* = 0.000) and homogeneity assumption (Levene *p* < 0.05). As the normality and homogeneity assumptions were not met for the general self-efficacy scale, a Kruskal‒Wallis test was employed to compare the self-efficacy levels of the participants across PA levels.

The Kruskal‒Wallis findings revealed a statistically significant difference (H(2) = 68.77, *p* = 0.000) in self-efficacy scale scores awarded by university students of differing PA levels. The data indicated that those with low PA levels (*n* = 559, rank mean = 1253.16) and moderate PA levels (*n* = 1101, rank mean = 1341.72) differed significantly from those with high PA levels (*n* = 1167, rank mean = 1559.23).

As a post-hoc analysis, Mann-Whitney U tests were conducted to determine which groups exhibited statistically significant differences. Given that the significance level of the Mann‒Whitney U tests was set at 0.05, a Bonferroni correction was applied by dividing the number of categories by 3, resulting in a significance level for all effects of 0.0167. The Bonferroni correction revealed no significant difference between the low PA levels (Mdn = 2.80) and the moderate PA levels (Mdn = 2.81) [U(N_low_=559, N_moderate_= 1101) = 2891, z=-2.019, *p* = 0.043]. Individuals in the low PA levels (Mdn = 2.80) presented lower self-efficacy scores than those in the high PA levels did (Mdn = 3.00) [U(N_Low=_559, N_high_= 1167) = 2548, z=-7.370, *p* = 0.000]. Individuals in the moderate-level PA levels (Mdn = 2.81) presented lower self-efficacy scores than did those in the high-level PA levels (Mdn = 3.00). The observed difference was statistically significant [U (_moderate_=11001, N_high_= 1167) = 5442, z=-6.308, *p* = 0.000].

A Mann‒Whitney U test was employed to evaluate the self-efficacy scale scores according to the gender of the participants. The findings revealed that male participants (Mdn = 3.00) presented higher self-efficacy scores than female participants did (Mdn = 2.80) [U(N_male_=1446, N_female_=1381) = 8673, z=-6.052, *P* = 0.000].

Furthermore, The Kruskal‒Wallis test findings revealed that there was no statistical difference (H(4) = 14.91, *p* = 0.05) between scores of the school year variable.

### Comparison of psychological resilience according to physical activity level, gender and school year

The mean scores for psychological resilience according to levels of PA were calculated. Individuals who were in the low PA level group presented a mean score of x̄= 3.14 (SD = 0.752), the moderate PA group presented a mean score of x̄= 3.30 (SD = 0.798), and the high PA group presented a mean score of x̄= 3.39 (SD = 0.775). Upon examination of the normality assumption of the scores awarded on the psychological resilience scale, it was determined that the calculated skewness (skewness = -0.27; standard error = 0.047) and kurtosis values (kurtosis = 0.592; standard error = 0 0.094) fell within the − 1 and + 1 range, by the guidelines [67]. Levene’s test yielded a p-value of 0.087 for the homogeneity assumption, indicating that the latter was satisfied. Given that the normality and homogeneity assumptions had been met, one-way analysis of variance (ANOVA) was employed to calculate the differences between the groups. The findings of the analysis indicated that there was a statistically significant discrepancy between the low, moderate, and high PA groups [F(2.2827) = 20.187, *p* = 0.000, η2 = 0.61]. As the requisite homogeneity assumption was satisfied, a post hoc Tukey-HSD analysis was employed to elucidate the discrepancies among the groups. The results demonstrated that individuals exhibiting a low level of PA (x̄= 3.14, SD = 0.752) demonstrated lower degrees of psychological resilience than individuals exhibiting a moderate level of PA (x̄=3.30 SD = 0.798) and individuals exhibiting a high level of PA (x̄ = 3.39 SD = 0.775). In other words, as the level of PA increased, an increase in psychological resilience scores was observed as well (See Table 3).

**Table 3:** Psychological resilience ANOVA findings

Psychological Resilience	Physical Activity Levels						*F*	*p*	*η2*
	Low		Moderate		High				
	$\bar{x}$	SD	$\bar{x}$	SD	$\bar{x}$	SD			
	3.14	0.752	3.30	0.798	3.39	0.775	20.187	0.000	0.61

The results of the independent sample t test investigating the discrepancy in psychological resilience levels between male and female participants revealed a statistically significant difference in psychological resilience scores between female (x̄=3.35, SD = 0.802) and male university students (x̄=3.26, SD = 0.764) [t(2829) = 4.160, *p* = 0.000, η2 = 0.052] (See Table 4).

**Table 4:** Psychological resilience scale t-test findings by gender variable

Psychological resilience	Gender							
	Female		Male					
	$\bar{x}$	SD	$\bar{x}$	SD	N	*t*	*p*	*η2*
	3.35	0.802	3.26	0.764	2829	4.160	0.000	0.052

A one-way analysis of variance was conducted to investigate the discrepancy in psychological resilience levels among participants stratified by their school year. The findings indicated that there was no statistically significant difference between the scores assigned to the psychological resilience scale across the school year variable [F(4.2825) = 1.191, *p* = 0.045] (See Table 5).

**Table 5:** Psychological resilience scale ANOVA findings by school year variable

Psychological resilience	School Year											
	Preparatory Year		1st Year		2nd Year		3rd Year		4th Year			
	$\bar{x}$	SD	$\bar{x}$	SD	$\bar{x}$	SD	$\bar{x}$	SD	$\bar{x}$	SD	*F*	*p*
	3.25	0.84	3.35	0.79	3.27	0.70	3.30	0.72	3.36	0.80	1.191	0.045

### Comparison of self-confidence according to physical activity level, gender and school year variables

When the normality assumption of the scores given to the participants’ self-confidence scale was examined, the skewness value (skewness =-0.556, standard error = 0.047) and kurtosis value (kurtosis = 1.067, standard error = 0.094) were calculated for the internal self-confidence subdimension. Skewness (skewness =-0.526, standard error = 0.047) and kurtosis (kurtosis = 1.126, standard error = 0.094) values were calculated for the external self-confidence subscale. These values are within a sufficient range to meet the normality assumption [67]. Levene’s statistic was used for the homogeneity assumption, and it was observed that the p value was greater than 0.05 for both subdimensions.

Given the normality and homogeneity assumptions inherent to the self-confidence scale data, one-way analysis of variance (ANOVA) was employed to ascertain whether there were any statistically significant differences in the self-confidence scale scores according to the PA level groups.

The findings of the ANOVA demonstrated a statistically significant difference between university students with low, moderate and high PA levels for the internal self-confidence subscale [F(2.2754) = 36.258, *p* = 0.000, η2 = 0.053]. An examination of the differences between the groups revealed that the mean internal self-confidence score of university students with low PA levels was 3.79 (SD = 0.65), whereas that of students with moderate levels of PA was 3.89 (SD = 0.63). For those with high levels of PA, the corresponding mean internal self-confidence score was 4.05 (SD = 0.65). As the findings indicate, a notable increase in internal self-confidence scores was observed for all groups as the level of PA increased.

For the external self-confidence dimension, ANOVA findings revealed that there was a statistically significant difference between university students with low, moderate and high PA levels [F(2.2786) = 30.720 *p* = 0.000 η2 = 0.051]. When the differences between the PA level groups were examined, the mean external self-confidence of the low PA level university students was 3.78 (SD = 0.66), the mean external self-confidence of the moderate PA level university students was 3.85 (SD = 0.64), and the mean external self-confidence of the high PA level university students was 4.01 (SD = 0.61). The results demonstrated that as the level of PA increased, there was a notable increase in external self-confidence scores across all groups, mirroring the trends observed in internal self-confidence (See Table 6).

**Table 6:** ANOVA results for comparison of PA levels by the subdimensions of the self-confidence scale

	Low		Moderate		High				
	$\bar{x}$	SD	$\bar{x}$	SD	$\bar{x}$	SD	*F*	*p*	*η2*
Internal Self-Confidence	3.79	0.65	3.89	0.63	4.05	0.60	36.258	0.000	0.053
External Self-Confidence	3.78	0.66	3.85	0.64	4.01	0.61	30.720	0.000	0.051

An independent sample t-test was employed to examine the differences in responses to the internal and external self-confidence subdimensions of the self-confidence scale, stratified by gender. The findings revealed that male participants presented significantly higher scores than female participants did [t(2918) = 3.518, *p* = 0.000, η2 = 0.004]. The mean score for male participants was 3.97 (SD = 0.64), whereas the mean score for female participants was 3.89 (SD = 0.61). With respect to the external self-confidence subscale, the findings indicated that male participants (x̄= 3.94, SD = 0.66) demonstrated statistically higher scores than female participants did (x̄= 3.86, SD = 0.63) [t(2918) = 3.710, *p* = 0.000, η2 = 0.004] (See Table 7).

**Table 7:** Self-confidence scale independent sample t-test findings by gender variable

	Gender						
	Female		Male				
	x̄	SD	x̄	SD	t	*p*	η2
Internal Self-Confidence	3.89	0.61	3.97	0.64	3.518	0.000	0.004
External Self-Confidence	3.86	0.63	3.94	0.66	3.710	0.000	0.004

**Table 8:** ANOVA findings of self-confidence scale by school year variable

	School Year											
	Preparatory Year		1^st^ Year		2^nd^ Year		3^rd^ Year		4^th^ Year			
	$\bar{x}$	SD	$\bar{x}$	SD	$\bar{x}$	SD	$\bar{x}$	SD	$\bar{x}$	SD	*F*	*p*
External Self-Confidence	3.91	0.64	3.97	0.61	3.89	0.61	3.88	0.66	4.04	0.62	2.527	0.069
External Self-Confidence	3.88	0.67	3.93	0.62	3.88	0.62	3.86	0.68	4.05	0.55	1.813	0.123

One-way analysis of variance was employed to ascertain whether there were any statistically significant differences in the participants’ responses to the internal and external self-confidence subdimensions of the self-confidence scale according to their grade level. The findings indicated that no statistically significant difference was observed between the school year variable and for either the internal self-confidence subdimension [F(4.2752) = 2.527, *p* = 0.069,] and the external self-confidence subdimension [F(4.2784) = 1.813, *p* = 0.123] (See Table 8).

## Discussion

The first aim of present study set out to explore how university students’ self-efficacy levels might differ depending on their physical activity (PA) levels, gender, and year of study. The findings indicated that university students who are in high level of PA group reported statistically higher self-efficacy score than their moderately or less active peers. These findings are supported by several studies from literature that suggesting being physically active support not only physical health but also well-being and self-perception [68, 69]. The relation between self-efficacy and PA can be explained by Bandura’s social cognitive theory [43]. The relationship between PA and self-efficacy can be explained by Bandura’s theory. Based on the theory, the experience of mastering skills through PA and the perception of competence are the main sources of self-efficacy. Current studies from literature suggesting that when people do regular PA and start to see progress or feel more capable, their confidence in themselves tends to grow as well [70].

Results also indicated that both moderate and low PA groups showed similar self-efficacy levels, the high activity group consistently reported higher scores. Based on this finding and similar studies it could be suggested that only sustained and intensive physical engagement might yield psychological gains beyond a certain threshold, a trend previously reported in youth and adult populations alike [71, 72].

Gender differences was also another notable variable. Findings indicated that male students’ self-efficacy score was significantly higher when compared to female students score. Supportive findings have been observed in various sport and education studies, where males often report greater self-efficacy, particularly in performance and achievement-related extent [73, 74]. Generally, males are often encouraged to be competitive and physically active, which can lead to stronger beliefs in their own abilities over time [75]. Cultural norms and socialization processes may play a role, as males are often encouraged to pursue and value physical and competitive experiences more than women, which can reinforce higher self-efficacy beliefs.

Moreover, no significant differences in self-efficacy were found across school years variable. While some literature has suggested that self-efficacy may develop with age and academic experience [76]. The relevant literature provides a variety of information on this topic. Some of studies showed that exist self-efficacy of students increase as the school years progressed, especially in school environment are more supportive like that encourage independence, offer meaningful feedback, and recognize their accomplishments [77]. However, some other studies emphasize that there is no significant change or even a decline in self-efficacy during later academic years, often due to increased academic pressure, competitive environments, or burnout [78].

Comparison of psychological resilience based on their PA level results indicated that participants with higher PA levels reported significantly greater psychological resilience compared to their low and moderately active counterparts. This aligns with previous research suggesting that engaging in regular PA not only improves physiological functioning but also enhances individuals’ capacity to cope with stress and adversity [68]. Literature also supports that individual who maintained an active lifestyle exhibited higher levels of resilience over time [79]. Similarly, a systematic review of multiple studies demonstrated the beneficial impact of exercise interventions on resilience in diverse populations, including adolescents and older adults [80, 81]. It is widely accepted that regular exercise has the capacity to stimulate the release of neurochemicals such as endorphins and serotonin, which have the potential to increase mood and resilience to stress. Furthermore, participation in PA has been linked to an enhanced sense of accomplishment and self-efficacy, which are crucial elements in the development of resilience. Moreover, PA provides individuals with opportunities to interact with one another and receive support, which can help mitigate the negative effects of stress and adversity. The present study adds to this growing body of evidence by demonstrating that even within a university student population, those engaging in high PA exhibit significantly higher resilience, highlighting the potential role of being physically active in higher education settings.

Regarding the gender variable, comparison of participants’ gender, female students scored slightly higher on psychological resilience than their male counterparts. This result of the study contrasts with some previous studies. While certain research suggests that males may demonstrate greater resilience due to socialized coping strategies [82], other studies propose that females’ greater emotional expression and stronger interpersonal support networks may positively influence resilience [83]. These findings imply that the relationship between gender and resilience may differ depending on the context and population, suggesting a need for further exploration of the sociocultural factors at play.

Notably, there were no significant differences in resilience scores across students from different school years. This might indicate that the academic experience throughout various years does not inherently affect resilience levels, or it could reflect the consistency of support systems and challenges encountered by students at different stages of university life. Hartley [84] observed similar patterns, noting that resilience tends to remain stable during college years unless influenced by specific interventions or significant life events. Additionally, it is possible that individual differences and external factors, such as family support, workload, or socioeconomic status, may have a more substantial impact on resilience than the school year itself.

The one of crucial finding of this study was the significant difference between PA level and both internal and external self-confidence. University students with higher PA levels reported significantly higher self-confidence scores, which is consistent with previous literature highlighting the positive effects of PA on psychological well-being. Ekeland [85] demonstrated that PA can enhance self-esteem and self-confidence, especially among adolescents and young adults.

Specifically, the internal self-confidence scores were highest among students with high PA levels, suggesting that PA contributes not only to external perceptions but also to an individual’s internal sense of self-efficacy and self-worth. This finding is in line with Harter’s [86] model of self-esteem, which posits that physical competence is a significant domain of self-esteem. Higher levels of PA may increase perceived physical competence, which, in turn, positively influences self-confidence [87]. One of the study conducted with university students demonstrated that participants who engaged in PA presented higher levels of self-confidence than those who did not [88] Similarly, it was found that university students who participated in a 12-week Latin dance training program exhibited marked increases in self-confidence scores relative to students who did not engage in Latin dance training [89].

Based on the gender variable analysis male participants scored statistically higher than females in both and external self-confidence subscales. These findings consistent with the several studies that investigated self-confidence differences among genders. Gender differences in self-confidence are primarily shaped by sociocultural and developmental influences rather than innate traits. According to Casale [90] the differences between societal norms for males and females play central role fort the development of self-confidence. Likewise, Casale’s study [90] Lirg et al., [91] and Hyde [92] found that men tend to exhibit higher levels of self-confidence across various domains, including physical and social contexts. This has been attributed to societal and cultural factors, where traditional gender roles often encourage men to demonstrate assertiveness and physical prowess, leading to higher self-esteem [93]. In sum, the results of our examination are consistent with the existing literature on the subject.

The last finding of the research was an analysis of whether the school year variable makes a difference in the self-confidence of university students. Although the analysis did not reveal statistically significant differences in self-confidence across different school year, subtle trends in the data suggest a gradual increase in both internal and external self-confidence as students’ progress through their academic journey. This pattern is consistent with research indicating that self-confidence tends to grow with academic experience, increased exposure to university life, and greater social integration [94]. According to Casale [90] self-confidence tends to increase with age and academic progression due to the accumulation of life experiences, improved self-regulation skills, and greater exposure to achievement-related situations. These findings support the idea that self-confidence is not fixed but evolves through contextual experiences and developmental stages during higher education.

### Limitations

The present study is confined to students enrolled in 10 distinct academic units situated within the central campus of Çanakkale Province. The study population was restricted to individuals between the ages of 17 and 30, and the data collection period was limited to three months. Moreover, since it was a cross-sectional design, so the findings of the research does not allow to establish cause and effect relationships between the variables. Furthermore, the sample group for this study was recruited only from one university students in Turkey, precluding the generalizability of the findings to different cultural contexts.

## Conclusion and recommendations

In conclusion, psychological factors, including self-efficacy, self-confidence and psychological resilience, which were the focus of our study conducted in the post pandemic period, play pivotal roles in mitigating the adverse effects on students. Considering the positive correlation between physical activity (PA) and the findings of our study, it is recommended that interviews be conducted with local administrations and other relevant organizations with the aim of enhancing the PA environments for students on and around the university campus. Furthermore, it would be beneficial to implement projects that encourage students to engage in PA and to determine policies that facilitate this.

### Impact on stakeholders

Moreover, the results of this study have the potential to benefit various stakeholders within the university setting. University administrations could use these findings to justify implementing more programs to encourage student participation towards PA, such as sports clubs, fitness workshops, or wellness days. For students, the research highlights how maintaining an active lifestyle may not only improve physical health but also support emotional resilience, confidence, and motivation. Units related to health, sports, or education can apply these findings when creating their programs or giving advice to their students. Mental health services within campuses may also consider including PA as part of stress management and personal development strategies. Beyond the campus, health promotion institutions can use these insights to shape campaigns that encourage PA to support mental well-being among young adults.

### Recommendations for future research

Finally, to build on the current study, future researchers are encouraged to broaden their sample population to include students from different universities, regions, and educational institutions. By including a more diverse population can make the findings more widely applicable. Longitudinal studies, which follow participants over a long period of time, could provide stronger evidence about if PA directly leads to improvements in self-confidence, resilience, and self-efficacy. Besides, using objective tools such as fitness trackers or mobile applications would improve the accuracy of data. Researchers should also consider examining specific types of PA to see if certain forms offer greater psychological benefits than others. It would also be valuable to include other related factors—such as diet, sleep quality, and social support—that may influence mental health. Furthermore, including open-ended interviews could provide a deeper understanding of how students personally relate to PA and mental well-being.

## Electronic supplementary material

Below is the link to the electronic supplementary material.

Supplementary Material 1

Supplementary Material 2

## Data Availability

The dataset used and analyzed during the current study is available from the corresponding author on reasonable request.
